# A new combinatorial megaplasmid library assembly method designed to screen for minimal pathways by using SCRaMbLE

**DOI:** 10.17912/micropub.biology.000657

**Published:** 2022-10-26

**Authors:** Wei Sheng Yap, Guillaume Thibault

**Affiliations:** 1 School of Biological Sciences, Nanyang Technological University, Singapore, 637551; 2 Current address: Department of Biochemistry, University of Toronto, Toronto, Canada; 3 Mechanobiology Institute, National University of Singapore, Singapore, 117411; 4 Institute of Molecular and Cell Biology, A*STAR, Singapore, 138673

## Abstract

Human proteins expressed in yeast are common to enhance protein production while the expression of functional human pathways remain challenging. Here, we propose a simple and economical high-throughput gene assembly method to create a yeast megaplasmid library from human cDNA to screen for minimal human functional pathways. We introduced artificial promoters followed by symmetric loxP sites into the megaplasmids using Golden Gate assembly coupled with streptavidin-bead-based purification. The isolated high molecular weight, randomly assembled cDNA megaplasmid library may be useful for high-throughput directed evolution experiments and may be adapted for use in other model organisms.

**Figure 1. A new combinatorial megaplasmid human cDNA library assembly method.**
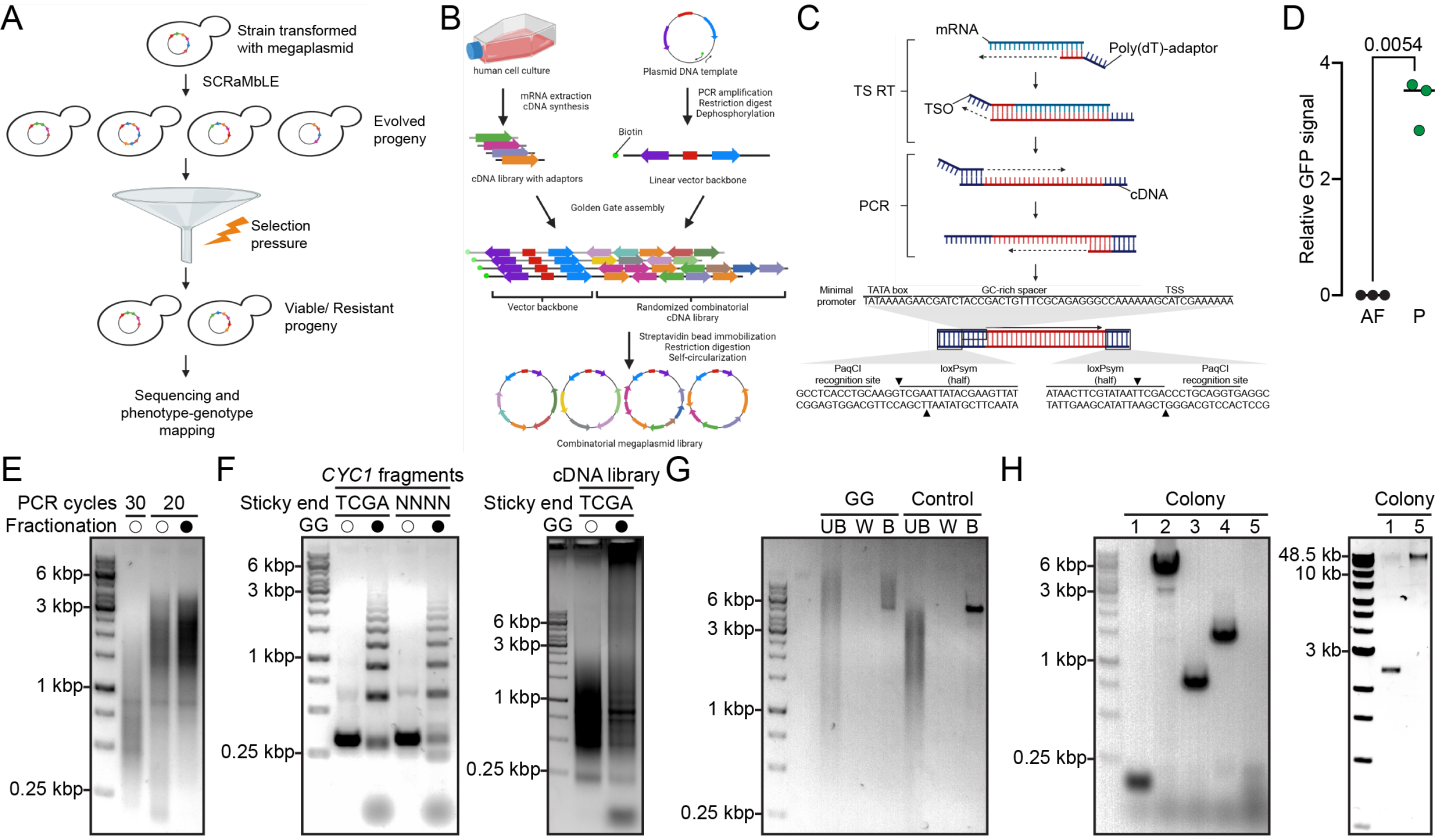
**A** , The potential use of the combinatorial megaplasmid library for directed evolution experiments using SCRaMbLE. **B** , An overview of the construction of the combinatorial megaplasmid library. **C** , Generation of synthetic minimal promoter-regulated cDNA expression library flanked by halves of loxPsym site and PaqCI restriction sites. TS RT, template-switching reverse transcription; TSO, template-switching oligos; dT, deoxythymidine; TSS, transcription start site. **D** , Validation of the synthetic minimal promoter (56 bp) activity in yeast. The GFP transcriptional fusion reporter of the synthetic minimal promoter (P) was quantified by flow cytometry. Fluorescence signals were normalized to autofluorescence (AF). **E** , Electrophoretic analysis of the cDNA PCR products. Partial cDNA synthesis is observed in first lane due to dNTP exhaustion. High-quality full-length cDNA was generated by reducing PCR cycles to 20 and followed by size fractionation (last lane). Size fractionated (closed circles) or non-fractionated (opened circles) samples are indicated. **F** , Proof of concept of concatemerization using Golden Gate assembly with PaqCI. GG, Golden Gate assembly. The presence (closed circles) or absence (opened circles) of the enzymes required for GG are indicated. **G,** Assembly of the biotinylated vector and the combinatorial cDNA library. Small aliquot of respective fractions was used for visualization on 0.6% agarose gel. Assembled megaplasmids were immobilized using streptavidin magnetic beads, followed by elution and SgrDI digestion. UB, unbound; W, wash; B, bound fractions. **H** , The assembled megaplasmids were self-circularized, electroeluted, and then transformed into *E. coli* by electroporation. The presence of the ligated inserts was verified by gel electrophoresis of colony PCR products (left panel) and/or extracted plasmids (right panel).

## Description

The laboratory model organism *S. cerevisiae* strain S288C has been used since the early nineteenth century and has become a powerful genetic tool ( Mortimer and Johnston, 1986 ). Despite being highly divergent, human and yeast share many conserved genes and pathways. Thus, it is unsurprising that many scientific breakthroughs are directly attributed to *S* . *cerevisiae* , including Nobel Prizes related to cell cycle regulators, telomeres, and autophagy. Subsequently, these discoveries were expanded in human and other higher eukaryotes. However, the complexity of the human genetic architecture remains a roadblock to new discoveries, including parallel pathways and a large proportion of unknown gene functions. Humanized yeast, a concept whereby human genes are expressed in yeast for further characterizations, has been widely practised in different degrees to study the complexity of the human system ( Kachroo et al., 2015 , Laurent et al., 2020 , Laurent et al., 2016 ). Many studies have expressed human orthologs to assess gene complementarity. Recently, with the advent of synthetic biology tools and pathway engineering, the entire human glycosylation pathway was engineered in yeast, signifying the modifiability and adaptability of yeast in modelling higher-order eukaryotes ( Hamilton et al., 2003 , Hamilton et al., 2006 ). However, this is possible only when a pathway is relatively well characterized ( Laurent et al., 2016 ). To this end, we propose a method combining bottom-up and top-down approaches to screen for the minimal human genes required to form a functional pathway in yeast.

We developed a method inspired by the recently ongoing synthetic yeast genome Sc2.0 project. The Sc2.0 project started with a bottom-up approach to build a synthetic chromosome containing only the nonredundant genome. In addition, a symmetric loxP site (loxPsym) was incorporated into the non-essential genomic regions between each coding sequence. This loxPsym feature is designed for the downstream directed evolution experiment via synthetic chromosome rearrangement and modification by loxPsym-mediated evolution (SCRaMbLE). SCRaMbLE is a powerful tool in the Sc2.0 project for many applications such as the creation of minimal cells and strains with desired phenotypes. Many studies that utilized SCRaMbLE have had high impact especially in the biotechnology field such as the generation of alkali and ethanol tolerance strains ( Ma et al., 2019 , Luo et al., 2018 ). SCRaMbLE was also used to expedite the identification of synthetic lethal interactions ( Wang et al., 2020 ). While the Sc2.0 project is indeed impressive, it is very costly due to the excessive reliance on gene synthesis and the tedious stepwise gene integration in yeast. Instead of building a whole chromosome out of synthesized gene fragments, we propose to apply SCRaMbLE to a yeast expression megaplasmid of randomly assembled human cDNA to identify the members required for a minimal pathway of desired phenotypes. This system is heterologous and therefore prevents the compensatory mechanisms that otherwise would occur in a same-species expression system. The megaplasmid can be maintained separately from the yeast genome and therefore eliminating the need for *in vivo* gene editing. We expect that upon completion of the megaplasmid library, it can be characterized genetically and phenotypically, and to be subjected to directed evolution to isolate strains with desired phenotypes (Fig. 1A).

We developed and optimized a blueprint of the megaplasmid library in which the vector backbone is a yeast centromeric shuttle vector and the inserts consist of the combinatorial human cDNA library (Fig. 1B). To construct the megaplasmid library, we first performed the template-switching reverse transcription for directional first strand cDNA synthesis which allowed the incorporation of the synthetic minimal promoter near the 5’ UTR of the human cDNA (Fig. 1C). The minimal promoter sequence was modified from a previously reported sequence ( Redden and Alper, 2015 ). We validated the promoter activity through the detection of a GFP reporter (Fig. 1D). To generate a representative and full-length double-stranded cDNA library, we amplified the first strand cDNA using low number of PCR cycles to avoid dNTP exhaustion. Next, we removed short cDNA fragments and other impurities by separating the PCR products on 1% agarose gel (Fig. 1E). To assemble the cDNA fragments into megachunks, we used Golden Gate assembly with PaqCI restriction enzyme which minimizes domestication issues seen in other commonly used enzymes that recognize hexanucleotide sequences. We first tested the assembly method using PCR-amplified fragments of a specific size, a 300 bp *CYC1* terminator sequence. We amplified the fragments using two different primer pairs in separate reactions so that the sticky ends produced by PaqCI digestion during Golden Gate assembly were four random nucleotides (NNNN) or TCGA overhangs, respectively. The rationale was to assess if the heterogeneity of sticky ends prevents self-circularization and promotes greater concatemerization. However, the same degree of concatemer formation was observed in both reactions, suggesting that the concatemerization ability was insignificantly influenced by the overhang mismatch (Fig. 1F, left panel). Given this insight, we proceeded with Golden Gate assembly of the cDNA library fragments using the “TCGA” overhang design. This approach should also generate the symmetric spacer sequence of the loxPsym site upon restriction digestion and ligation. As expected, high molecular weight concatemers of the cDNA library fragments were observed after Golden Gate assembly (Fig. 1F, right panel). Next, we prepared the vector backbone by linearizing the centromeric plasmid pGT731 by PCR where one of the primers was 5’-end-biotinylated for subsequent immobilization steps (Fig. 1B). The non-biotinylated end of the backbone vector was cleaved by the restriction enzyme XhoI to produce a sticky end compatible to the “TCGA” overhang produced by the PaqCI digestion. Next, we performed Golden Gate assembly of the vector and the loxPsym-engineered cDNA library, followed by pulldown of the assembled megaplasmid using streptavidin magnetic beads. A distinctive band of the immobilized vector at ~5 kbp was observed in the bound fraction of the negative control (without PaqCI and T4 Ligase), whereas the band corresponding to the vector in the Golden Gate assembly reaction smeared and shifted upwards, indicating successful ligation of the heterogenous cDNA library fragments with the vector (Fig. 1G). The assembled and immobilized DNA products were digested with SgrDI to cut near the biotinylated ends and to generate compatible sticky ends. The released DNA products were self-circularized by T4 ligase and separated on gel electrophoresis for extraction of high molecular weight megaplasmids by electroelution. We transformed the megaplasmids into *E. coli* for propagation. Probably owing to the cryptic sites and the repetitive sequences within the cDNA library, the colonies harbouring the megaplasmids were relatively smaller in size. Using a pair of vector-specific primers, we amplified the cDNA library inserts by colony PCR to validate the ligation product. The gel electrophoresis revealed the presence (colony 2, 3 and 4) or absence (colony 1) of ligated inserts (Fig. 1H). Intriguingly, colony 5 showed neither the empty vector band nor an upshifted band indicating the presence of an insert. We hypothesized that it could be due to the presence of a huge chunk of insert which is beyond the amplification capability of a conventional PCR. Therefore, we extracted the plasmids from colony 1 and 5 to compare their sizes using gel electrophoresis. Indeed, we noticed the presence of a megaplasmid in colony 5. It is noteworthy that the megaplasmid is in circular and possibly supercoiled form, suggesting that the megaplasmid may be well over 50 kbp in size.

Historically, DNA shuffling techniques were employed for the evolution of single known protein coding sequences, *de novo* chimeric protein construction, and peptide aptamer screening ( Stemmer, 1994b , Stemmer, 1994a , Fujishima et al., 2015 ). Here, we applied the DNA shuffling technique to express a pool of human proteins in yeast for screening of minimal pathways. Our work demonstrates a streamlined method to create a randomized combinatorial megaplasmid library. In synthetic biology, creating a synthetic chromosome is technically challenging. The Sc2.0 project created the synthetic chromosomes by replicating the essential sequences from the yeast genome. In our system, replicating the human genome to be used directly in yeast is not feasible due to the incompatibility of the transcriptional machinery. Instead, we designed a synthetic minimal promoter for heterologous expression to minimize genome instability as a result of repetitive sequences. A better approach is to diversify the short promoter sequences, particularly the spacer regions, although it may incur a higher cost from gene synthesis ( Kotopka and Smolke, 2020 ). The heterologous expression of human proteins in yeast may be toxic especially in high concentration. Therefore, the addition of a controllable cis-regulatory transcriptional silencer may be advantageous.

The assembly of a megaplasmid is extremely challenging considering the scale of the molecular construction. Golden Gate assembly was chosen for seamless ligation among the cDNA fragments and between the ligated cDNA fragments and the destination vector. The linearized vector was biotinylated at one end and restriction digested on the other to prevent self-circularization and promote concatenation. Moreover, the biotin conjugated to the vector allowed bead-based purification which removed most of the unligated cDNA fragments that interfere with the electrophoretic analysis, facilitating the extraction of the high molecular weight megaplasmid during the next step. In conclusion, this bottom-up assembly method presented a cheap alternative for the construction of combinatorial library of high complexity. Moreover, the complexity of the library can be easily manipulated, for instance, by transforming *S. cerevisiae* with multiple megaplasmids of different markers. This method can possibly be modified and adapted for use in other model organisms.

## Methods

**Strains.** *S. cerevisiae* wild-type (WT) strain background BY4741 ( *his3Δ1 leu2Δ0 met15Δ0 ura3Δ0* ) was used in this study. In Figure 1D, WT strain was transformed with empty plasmid pGT562 or GFP reporter plasmid pGT784, respectively, using standard lithium acetate transformation protocols.

**Plasmids used in this study.** Plasmids used in this study are listed in Table 1. Plasmids were constructed either by restriction or Gibson cloning. The pGT731 plasmid was cloned by inserting an additional SgrDI restriction site into a SacI/XbaI double digested pRS416 vector. The GFP reporter plasmid, pGT784, was cloned by the assembly of the pGT570 backbone and the synthetic minimal promoter insert. The backbone was amplified from pGT570 using WS170 primer pair to remove the promoter, whereas the synthetic minimal promoter insert was amplified from the cDNA library using TATA-TSS_F and TSS_R primer pair.

**Flow cytometry.** Yeast cells expressing either the synthetic-minimal-promoter-driven GFP or the empty vector control were grown to mid-log phase at 30ºC. The fluorescence intensity was measured using the LSRFortessa X-20 (BD) flow cytometer by exciting at 488 nm and collecting through a 505 nm longpass filter and a 530/30 bandpass filter. A median readout from 10,000 cells was acquired via the software FACSDiVA v 8.0. Data was analysed with the software FlowJo 10.8.0. Reported GFP fluorescence levels were normalized to autofluorescence from the empty vector control.

**Human cDNA yeast expression library preparation.** Total human mRNA was extracted from RPE-1 cells using NEB Magnetic mRNA Isolation Kit (NEB S1550S). Extracted mRNA was used for cDNA synthesis by template-switching reverse transcription (NEB M0466). The oligo (dT)18 primer, WS220, used during the reverse transcription contains an adaptor region of half a loxPsym site and a PaqCI restriction site whereas the template-switching primer, TSO contains the full sequence of the synthetic minimal promoter (Fig. 1C). The cDNA products were treated with Exonuclease I to remove residual primers, and then amplified using WS219 and WS220 primers which added half of a loxPsym site and a PaqCI restriction site to the 5’ end of the products. The amplified cDNA library was column purified (Biobasic BS664) and ran through the size exclusion spin-column (Takara Bio Inc. 636079) for size fractionation.

**Megaplasmid assembly and purification.** To prepare the vector backbone, pGT731 was amplified with WS165 primer pair and column purified. The purified backbone was then incubated with XhoI, DpnI, and CIP for restriction digestion and dephosphorylation. The digested backbone was column-purified again. The backbone was combined with the cDNA library at a 1:5 mass ratio for Golden Gate assembly with PaqCI restriction enzyme (NEB R0745). The Golden Gate assembly reaction was set up according to the manufacturer’s protocol. After the assembly, the linear DNA plasmid was captured by the streptavidin-conjugated magnetic beads (Invitrogen 11206D). The beads were reconstituted with the binding and washing (BW) buffer (5 mM Tris-HCl, pH 7.5, 0.5 mM EDTA, 1 M NaCl) prior to DNA coupling. The DNA-bound beads were washed twice with BW buffer before reconstituting in buffer R (Thermo Scientific BR5). After equilibrating in buffer R, the coupled plasmid DNA was released by SgrDI restriction digestion (Thermo Scientific ER2031). The released plasmid DNA was self-circularized by the addition of T4 DNA ligase (NEB M0202) and ATP (NEB P0756) according to the manufacturer’s protocol. The circular plasmid DNA was run on a 0.6% agarose gel electrophoresis for extraction of high molecular weight megaplasmids by electroelution (G-Biosciences 786-001).

**Bacterial transformation by electroporation.** The electroeluted megaplasmids were transformed into Stable Competent *E. coli* (NEB C3040H) by electroporation. In brief, 1 µl of the megaplasmids was added to 50 µl of electrocompetent cells and mixed on ice. The mixture was transferred to a pre-chilled 0.2 cm gap width electroporation cuvette (Bio-Rad 1652086) and pulsed at 2.5 kV with the Bio-Rad Gene Pulser Xcell Electroporation System. The electroporated cells were resuspended in 1 ml of outgrowth medium and recovered for 1 h at 37ºC. Cells were then plated on ampicillin selection plates and incubated at room temperature until the appearance of colonies.

**Colony PCR.** The *E. coli* transformants of relatively smaller colony size were picked and transferred to individual PCR reaction mix. The vector-specific universal primers of T3 and T7 promoters were used for the PCR amplification. The PCR products were then run on a 0.8% agarose gel electrophoresis and visualized afterwards. The presence of a ~170 bp band indicates an empty vector, whereas the presence of an upshifted band indicates the presence of ligated inserts.

**Megaplasmid extraction from *E. coli.*** *E. coli* transformants were each inoculated in a 3 ml selective medium and grown at room temperature for 2 days. Alkaline lysis was performed using the reagents provided by the plasmid DNA miniprep kit (Biobasic BS614) according to the manufacturer’s protocol. After the neutralization step, the lysate was clarified by centrifugation and the removal of the pellet fraction. The clarified lysate containing the plasmid DNA was precipitated by the addition of an equal volume of pure isopropanol and centrifugation. The pellet was washed twice and air-dried. Finally, the pellet was resuspended gently in elution buffer using a cut pipette tip.

Table 1. **Plasmids used in this study**

**Table:** 

Plasmid	Encoded protein	Promoter	Vector	Source
pGT562	-	-	pRS415	This study
pGT570	GFP	*GCN4*	pRS415	( Yap and Thibault, 2022 )
pGT731	-	-	pRS416	This study
pGT784	GFP	*TATA-TSS*	pRS415	This study

Table 2. **Oligonucleotide** **Primers used in this study**

**Table:** 

Primer	Sequence (5’ to 3’)
TSO	Biotin-TATAAAAGAACGATCTACCGACTGTTTCGCAGAGGGCCAAAAAAGCATCGAAAAAAGGGG
WS219	GCCTCACCTGCAAGGTCGAATTATACGAAGTTATTATAAAAGAACGATCTACCG
WS220	GCCTCACCTGCAGGGTCGAATTATACGAAGTTATTTTTTTTTTTTTTTTTT
PaqCI(TCGA)-CYC1t_F	GCCTCACCTGCAAGTTCGAACAGGCCCCTTTTCCTTTGT
PaqCI(TCGA)-CYC1t_R	GCCTCACCTGCAGGTTCGAGATGAGAGTGTAAACTGCGA
PaqCI(NNNN)-CYC1t_F	GCCTCACCTGCAAGTNNNNACAGGCCCCTTTTCCTTTGT
PaqCI(NNNN)-CYC1t_R	GCCTCACCTGCAGGTNNNNGATGAGAGTGTAAACTGCGA
WS165F	Biotin-GACTTCTAGAGCGGCCGCCACCG
WS165R	GCCTACTAGTGGATCCCCCGGGCT
WS170F	ATGAGTAAAGGAGAAGAACTTTTCACTGG
WS170R	CTGCAGGAATTCGATATCAAGCTTATCG
TSS_R	TCCTTTACTCATTTTTTTCGATGCTTTTTTGGC
TATA-TSS_F	CGAATTCCTGCAGTATAAAAGAACGATCTACCGACTG
T3 promoter	GCAATTAACCCTCACTAAAGG
T7 promoter	TAATACGACTCACTATAGGG
